# Reprogramming of aerobic glycolysis in non‐transformed mouse liver with pyruvate dehydrogenase complex deficiency

**DOI:** 10.14814/phy2.14684

**Published:** 2021-01-05

**Authors:** Mulchand S. Patel, Saleh Mahmood, Jiwon Jung, Todd C. Rideout

**Affiliations:** ^1^ Department of Biochemistry Jacobs School of Medicine and Biomedical Sciences University at Buffalo Buffalo NY USA; ^2^ Department of Exercise and Nutrition Sciences School of Public Health and Health Professions University at Buffalo Buffalo NY USA

**Keywords:** aerobic glycolysis, Liver PDC deficiency, PKM2 and LDHB gene expression, SIRT1 down‐regulation

## Abstract

The Pyruvate Dehydrogenase Complex (PDC), a key enzyme in glucose metabolism, catalyzes an irreversible oxidative decarboxylation reaction of pyruvate to acetyl‐CoA, linking the cytosolic glycolytic pathway to mitochondrial tricarboxylic acid cycle and oxidative phosphorylation. Earlier we reported a down‐regulation of several key hepatic lipogenic enzymes and their upstream regulators in liver‐specific PDC‐deficient mouse (L‐PDCKO model by deleting the *Pdha1* gene). In this study we investigated gene expression profiles of key glycolytic enzymes and other proteins that respond to various metabolic stresses in liver from L‐PDCKO mice. Transcripts of several, such as hexokinase 2, phosphoglycerate kinase 1, pyruvate kinase muscle‐type 2, and lactate dehydrogenase B as well as those for the nonglycolysis‐related proteins, CD‐36, C/EBP homologous protein, and peroxisome proliferator‐activated receptor γ, were up‐regulated in L‐PDCKO liver whereas hypoxia‐induced factor‐1α, pyruvate dehydrogenase kinase 1 and Sirtuin 1 transcripts were down‐regulated. The protein levels of pyruvate kinase muscle‐type 2 and lactate dehydrogenase B were increased whereas that of lactate dehydrogenase A was decreased in PDC‐deficient mouse liver. Analysis of endoplasmic reticulum and oxidative stress indicators suggests that the L‐PDCKO liver showed evidence of the former but not the latter. These findings indicate that (i) liver‐specific PDC deficiency is sufficient to induce “aerobic glycolysis characteristic” in mouse liver, and (ii) the mechanism(s) responsible for these changes appears distinct from that which induces the Warburg effect in some cancer cells.

## Graphical abstract

In the present study we investigated gene expression profiles of key glycolytic enzymes and other proteins that respond to various metabolic stresses in liver from L‐PDCKO mice. Our findings indicate that (i) liver‐specific PDC deficiency is sufficient to induce ‘aerobic glycolysis characteristic’ in mouse liver, and (ii) the mechanism(s) responsible for these changes appears distinct from that which induces the Warburg effect in some cancer cells.

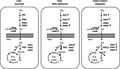

## 1 INTRODUCTION

Cancer cells exhibit atypical adaptive metabolic characteristics in the oxidative metabolism of glucose depending upon the origin of cell‐type and growth patterns (Goetzman & Prochownik, 2018; Vander Heiden et al., 2009). Cancers originating from prostate, lung and liver show increased mitochondrial metabolism of pyruvate derived from glucose with increased gene expression as well as increased functional activity of the pyruvate dehydrogenase complex (PDC) (Chen et al., 2018; Dolezal et al., 2017; Marin‐Valencia et al., 2012). These changes not only support increased demand for ATP generation by mitochondrial oxidative phosphorylation for biosynthetic processes for rapidly growing cancers but also provide acetyl‐CoA as the precursor for biosynthesis of lipids such as fatty acids and cholesterol for rapidly proliferating cells (Chen et al., 2018). Additionally, pyruvate carboxylase‐mediated anaplerosis supports survival and proliferation of some cancers and rapidly growing nontransformed fibroblasts (Sellers et al., 2015; Wang et al., 2019).

In contrast, in other cancers originating from lung and intestine, hypoxia‐induced factor‐1α (HIF‐1α), together with the expression of specific oncogene kinases, cause alterations in gene expression profiles that correspond to an aerobic glycolytic metabolism of glucose with reduced mitochondrial oxidation of glucose‐derived intermediate such as pyruvate (classically known as the Warburg effect) (DeBerardinis et al., 2008; Papandreou et al., 2006). PDC, a multienzyme complex, is the gatekeeper of pyruvate oxidation in the mitochondria, and its first catalytic component, the pyruvate dehydrogenase (PDH), is subject to inactivation by phosphorylation of three specific serine residues in its α subunit by a family of four dedicated PDH kinases (PDK 1–4) (Bowker‐Kinley et al., 1998; Korotchkina & Patel, 2001; Patel & Korotchkina, 2003). Different PDKs are up‐regulated in specific cell and cancer‐dependent patterns, causing increased inactivation of PDH and the PDC complex as a whole (Dupuy et al., 2015; Grassian et al., 2011; Kim et al., 2006; Saunier et al., 2016; Woolbright et al., 2019). Expression of specific oncogene kinases (FGFR1, FLT3, BCR‐ABL) in cancer cells also phosphorylate specific tyrosine residues in several PDC component proteins, promoting PDC inactivation (Fan et al., 2014; Hitosugi et al., 2011; Shan et al., 2014). Furthermore, HIF‐1α up‐regulates PDK1 gene expression to enhance PDK‐dependent inhibition of PDH (Kim et al., (2006)) and HIF‐1α also down‐regulates mitochondrial oxidative phosphorylation system (Reznik et al. 2017). Collectively, these “adaptive” changes due to the Warburg effect result in the constitutively increased glycolytic metabolism needed for rapid cytosolic ATP generation, the anabolic substrates needed for the accrual of biomass and the production of pyruvate‐derived lactate, which regenerates the NAD^+^ needed to sustain the entire glycolytic pathway. Increases in the expression of several key glycolytic enzymes such as hexokinase 2 (HK2), pyruvate kinase muscle isozyme‐2 (PKM2) and lactate dehydrogenase type A (LDHA) (Dang et al., 2008; DeBerardinis et al., 2008; Lewis et al., 2000) are well‐documented in cancers exposed to hypoxic conditions. The mitochondrial pyruvate carrier (MPC), a multienzyme complex composed of two distinct MPC1 and MPC2 subunits, is required for mitochondrial pyruvate import (Schell & Rutter, 2013). Under‐expression or deletion of *MPC* genes, particularly *MPC1*, is observed in many cancers with poor prognosis (Eboli et al., 1977; Paradies et al., 1983; Schell et al., 2014).

It is not clear whether the above “adaptive” metabolic changes are acquired as a result of the constraints of a tumor microenvironment or are necessary prerequisites for its initiation. Using the earliest transition of normal human intestinal epithelium cells to hyperplastic adenoma, Bensard et al. (2020) recently showed that the earliest stages of colorectal cancer initiation were supported by aerobic glycolytic metabolism coupled with down‐regulation of the mitochondrial pyruvate carrier (MPC). Additionally, using genetically altered MPC in Drosophila and mouse models, these investigators demonstrated that reduction in mitochondrial pyruvate metabolism due to MPC depletion was sufficient to enhance oncogenic susceptibility of intestinal cells from both species (Bensard et al., 2020).

Mitochondrial pyruvate metabolism is carried out by two principal enzymes, namely the PDC and pyruvate carboxylase (PC), generating acetyl‐CoA and oxaloacetate respectively, to support the formation of the tricarboxylic acid (TCA) cycle intermediates for the oxidative metabolism as well as to supply intermediates for the biosynthetic processes in the cell. Since genetic elimination of the MPC affects the functions of both PDC and PC, it is difficult to access their relative contributions to the development of the metabolic shift (aerobic glycolysis) in otherwise normal intestinal cells exposed to normal oxygen level. To evaluate the specific contribution of PDC in this ‘adaptive’ metabolic process, we employed a liver‐specific *Pdha1*‐knockout mouse model (with PDC activity deficiency) to evaluate its impact on a liver‐specific metabolic shift in the glycolytic gene expression profile. Our findings clearly show that hepatocyte‐specific PDC loss is sufficient to alter the expression of several key glycolytic enzymes, thus mimicking the aerobic glycolytic response seen in many cancer cells.

## 2 METHODS

### 2.1 Generation of liver‐specific L‐PDCKO mice

Mice (129 J genetic background) harboring the *Pdha1*‐*flox8* allele(s) were generated as described previously (Johnson et al., 2001), and their genetic background was then switched to B6 by back‐crossing of floxed females with B6 wild‐type males for 10 generations (Patel et al., 2014). The progeny of the last breeding was intrabred to derive a *Pdha1*‐floxed colony with the B6 genetic background. To delete exon 8 in the *Pdha1* gene in the liver from male progeny (L‐PDCKO), homozygous *Pdha1^flox8^* ^/^ *^flox8^* females with the B6 genetic background (Patel et al., 2014) were bred with the C57BL/6‐TgN9AlbCre 21Mgn transgenic male mice (The Jackson Laboratory), carrying an autosomally integrated *Cre* gene driven by the albumin promoter (Postic et al., 1999). The *Pdha1* gene is localized on chromosome X in the mouse, and hence the deletion of its exon 8 by Cre recombinase created complete deletion of PDHα protein in the male progeny only. Since female progeny were heterozygous for the *Pdha* gene expression in the liver, we did not include them in this study. To generate Cre‐positive control male mice (L‐PDCCT), wild‐type B6 females were bred with transgenic albumin promoter‐driven‐*Cre* B6 males. Mice had free access to a standard rodent chow diet and water. Tail DNA from ~12‐day‐old progeny were isolated using a kit (OmniprepTM I; Genotechnolgy) and genotyped (Mahmood et al., 2016). Only male mice were weaned on postnatal day 21 on a standard rodent chow diet and water *ad libitum* and investigated in this study. Approximately 2‐month‐old L‐PDCCT and L‐PDCKO male mice in the fed state were deeply anesthetized, livers were quickly removed, frozen in liquid nitrogen, and stored at −80°C. The procedures for breeding and maintenance of all mouse colonies and all experiments were performed in accordance with the Guide for the Use and Care of Laboratory Animals and approved by the Institutional Animal Care and Use Committee of the University at Buffalo (Protocol #201900008).

### 2.2 Quantitative real‐time polymerase chain reaction (qRT‐PCR) for gene expression

Livers (~100 mg) were homogenized in TRIzol reagent to extract total RNA as per the manufacturer's instructions (Life Technologies). Total RNA (~1 µg) was reverse transcribed into cDNA using an iScript cDNA kit (Bio‐Rad). RT‐PCR reactions were performed using appropriately diluted cDNA in triplicate with (β‐actin) serving as an internal control, and gene expression levels were quantified using a CFX96 Touch RT‐PCR detection system (Bio‐Rad) according to the manufacturer's recommendation. Relative quantification of amplified DNA was performed using the delta‐delta Ct method (Choi et al., 2010; Mahmood et al., 2016). Primers used for gene expression analysis are presented in Table S1 (https://figshare.com/articles/figure/_/12821813).

### 2.3 Western blotting

Liver homogenates were fractionated according to the procedure described earlier (Mahmood et al., 2016). Briefly, liver (~100 mg) was homogenized in buffered sucrose containing a protease inhibitor cocktail (Sigma‐Aldrich) and subjected to centrifugation to obtain the cytosolic fraction (Mahmood et al., 2016), and stored at −80^0^C. The protein content was determined using Bio‐Rad protein assay. Cytosolic proteins were separated and immunodetected using the Western blotting technique as described previously (Mahmood et al., 2016). Equal amounts of protein (ranging from 25 to 50 µg) were separated using sodium dodecyl sulfate‐polyacrylamide gel electrophoresis, transferred to nitrocellulose membranes, and detected using specified antibodies as listed: PKM1 (Cell Signaling, #7067S; dil:1:1000), PKM2 (Cell Signaling #4053S; dil:1:1000), LDHA (Cell Signaling, #2012S; dil:1:1000), LDHB (Invitrogen #PA5‐85883; dil:1:3000), and anti‐PDH antibody (dil:1:2000) (Mahmood et al., 2016) to detect pyruvate dehydrogenase component of PDC. β‐Actin used as a loading control was detected using β‐actin (D6A8) antibody (Cell Signaling, #8457S; dil:1;1000). Protein bands were visualized using an Enhanced Chemiluminescence kit (Perkin‐Elmer) and analyzed using Bio‐Rad ChemiDoc MP image analyzer.

### 2.4 Data analysis

Results are expressed as means ±SE of 5 to 8 animals as indicated, and statistical differences between the means were accessed using Students’ t‐test and a significant difference was assigned when the *p* value was <0.05.

## 3 RESULTS

### 3.1 Analyses of Pdha1mRNA and PDH protein in liver‐specific PDC‐deficient (L‐PDCKO) male mice

Progeny from the breeding of *Pdha1*‐floxed homozygous females with liver‐specific albumin‐Cre transgenic males were found to be normal in average litter size with no embryonic lethality and their postnatal growth was similar to that of the control L‐PDCCT progeny (results not shown), as reported previously (Mahmood et al., 2016). A detailed genomic analysis of L‐PDCKO and L‐PDCCT mice was reported previously (Mahmood et al., 2016) and similar genotypic results were observed for the mice reported here. Genomic analysis of tail DNA showed the presence of the 800 bp *Pdha1^flox^* allele and the 240 bp *Cre* allele in L‐PDCKO male mice and the presence of the 700 bp Pdha1^wt^ allele and the 240 bp *Cre* allele in L‐PDCCT male mice. Liver genomic DNA analysis of 2‐month‐old L‐PDCKO male mice showed the presence of the 400 bp *Pdha* ^Δex8^ deleted allele, whereas the presence of the 800 bp *Pdha1*‐floxed allele was detected in skeletal muscle and heart, indicating deletion of the *Pdha1* allele in the liver only (results not shown), as reported previously (Mahmood et al., 2016). These tissues from the age‐matched control L‐PDCCT male mice showed the presence of the 800 bp floxed‐*Pdha1* allele as reported previously (Mahmood et al., 2016). The presence of the 240 bp *Cre* allele was detected in all tissues examined from both the L‐PDCCT and L‐PDCKO male mice, indicating the ubiquitous presence of the *Cre* transgene in all tissues analyzed. As expected, using western blot analysis the complete absence of the α as well as β subunits of the PDH component of PDC was detected in the liver of L‐PDCKO male mice compared with livers from L‐PDCCT mice, and both the subunits of PDH were present in all other tissues analyzed in both the groups of mice (Mahmood et al., 2016). In the absence of the α subunit of PDH, the β subunit of PDH was found to be absent due to its instability (Ho et al., 1986).

### 3.2 Hepatic gene expression of key glycolytic enzymes in L‐PDCKO male mice

**Figure 1:**
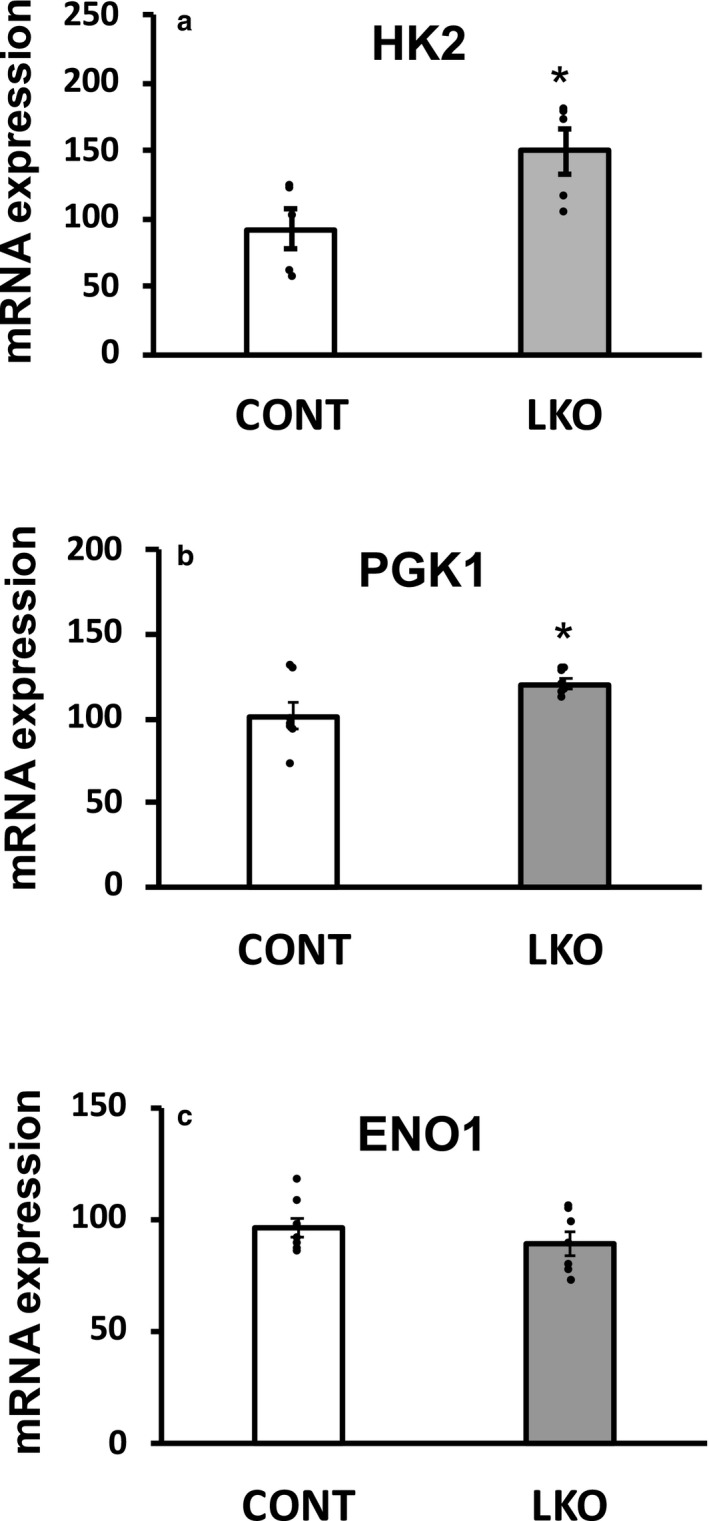
Quantitative real‐time PCR analysis of mRNA expression of three glycolytic enzymes (a) Hexokinase 2 (HK2; gene *Hk2*), (b) Phosphoglycerate kinase 1 (PGK1; *Pgk1*), and (c) Enolase 1 (ENO1; *Eno1*) in livers from control (L‐PDCCT) and liver‐specific PDC knockout (L‐PDCKO) mice. CONT: control; LKO: liver‐knockout. The results are means ±SE (*n* = 5–7). * *p* < 0.05

**Figure 2:**
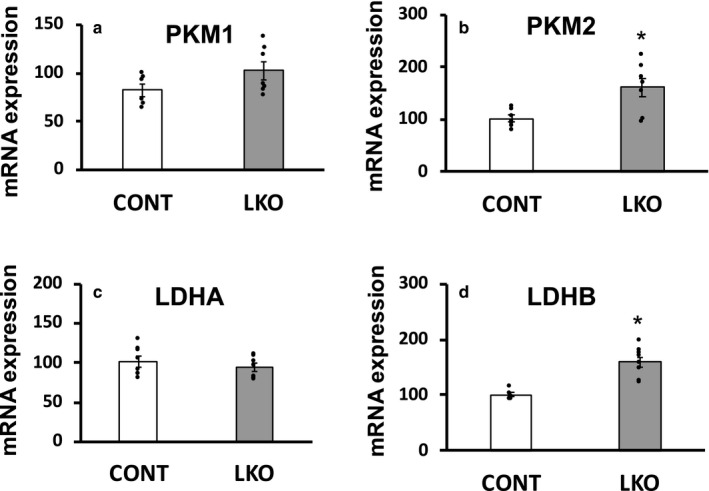
Quantitative real‐time PCR analysis of mRNA expression of four glycolytic enzymes (a) Pyruvate kinase M1 (PKM1; gene *Pkm1*), (b) Pyruvate kinase M2 (PKM2; *Pkm2*), (c) Lactate dehydrogenase A (LDHA; *Ldha*) and (d) Lactate dehydrogenase B (LDHB; *Ldhb*) in livers from control L‐PDCCT (CONT) and L‐PDCKO (LKO) mice. The results are means ±SE (*n* = 5–8). *p < 0.05

Based on the well‐characterized metabolic switch for aerobic glycolysis, classically known as the Warburg effect, in many types of cancer cells, we focused on “adaptive changes” in hepatic gene expression of several key enzymes in the glycolytic pathway in the liver of L‐PDCKO mice. We performed qRT‐PCR analyses of several key glycolytic genes such as hexokinase 2 (HK2; gene symbol *Hk2*), phosphoglycerate kinase 1 (PGK1; *Pgk1*), enolase 1 (ENO1; *Enol*), pyruvate kinase muscle‐type 1 and 2 (PKM1 and 2; *Pkm1* and *Pkm2*), and lactate dehydrogenase A and B (LDH A and B; *Ldha* and *Ldhb*). Significant increases in gene expression of *HK2* and *PGK1* were observed in the liver of L‐PDCKO mice compared with the control L‐PDCCT mice (Figure 1a, b). The expression of hepatic *Enol* gene was not significantly affected in PDCKO mice (Figure 1c). Expression of two enzymes involved in the generation of pyruvate by pyruvate kinase (PK) and its reduction to lactate by LDH are highly up‐regulated to support aerobic glycolysis in many different cancer cell‐types. Normal liver expresses liver‐specific pyruvate kinase (PKL) isozyme with low level expression of muscle‐specific PK isozymes (PKM1 and PKM2) resulting from an alternative splicing of the *Pkm* gene. Down‐regulation of liver‐specific PKL (*Pklr*) gene expression in L‐PDCKO was reported earlier (Mahmood et al., 2016). The expression of the *Pkm1* and *Pkm2* genes was differentially affected in the liver of L‐PDCKO mice (Figure 2). Hepatic expression of the *Pkm1* gene was not significantly altered but that of the *Pkm2* gene was significantly up‐regulated in L‐PDCKO mice compared with L‐PDCCT mice (Figure 2a, b). Similarly, expression of the *Ldha* gene remained unaffected whereas that of the *Ldhb* gene was significantly up‐regulated in the liver of L‐PDCKO mice compared with the L‐PDCCT mice (Figure 2c, d).

### 3.3 Hepatic protein levels of key glycolytic enzymes in L‐PDCKO mice

**Figure 3:**
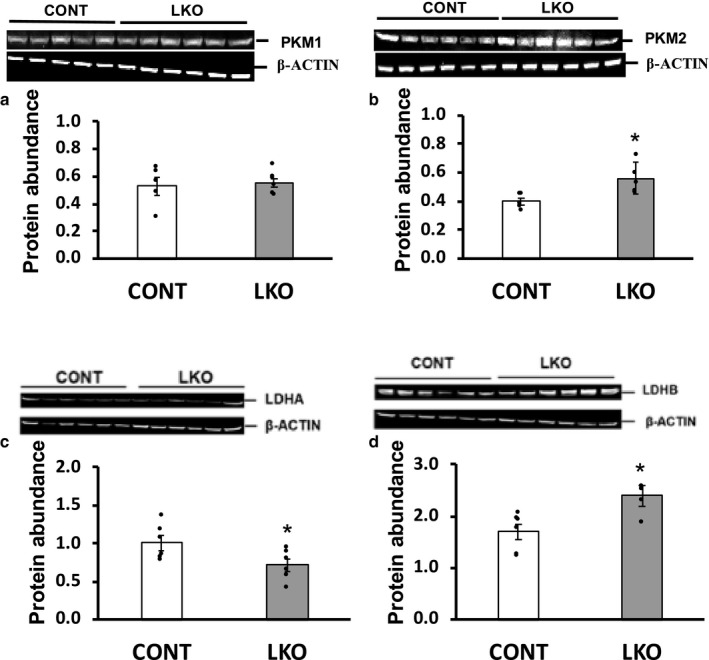
Western blot analysis of liver glycolytic enzymes. (a) Pyruvate kinase M1 (PKM1), (b) Pyruvate kinase M2 (PKM2), (c) Lactate dehydrogenase A (LDHA) and (d) Lactate dehydrogenase B (LDHB) in livers from control L‐PDCCT (CONT) and L‐PDCKO (LKO) mice. β‐actin was used as an internal control. The results are means ±SE (*n* = 5–6). * *p* < 0.05. Relative protein expression level was quantified and reported in a bar graph form

To further evaluate the possible effect of differential expression of the *Pkm* and *Ldh* genes on their encoded proteins, we performed western blot analyses using isozyme‐specific antibodies. Compared to L‐PDCCT mice, PKM1 protein levels in the livers of L‐PDCKO mice were not significantly altered whereas that PKM2 protein levels were significantly increased (Figure 3a, b). These findings were consistent with their gene expression profiles reported above (see Figure 2a, b). LDHA protein was significantly decreased in L‐PDCKO livers (Figure 3c) despite there being no significant changes in its transcripts c (Figure 2c). Hepatic level of LDHB protein was significantly increased in L‐PDCKO mice compared with L‐PDCCT mice (Figure 3d). This finding is supported by up‐regulation of the *Ldhb* gene in L‐PDCKO mice (Figure 2d).

### 3.4 Hepatic gene expression of HIF‐1α and other metabolic and cellular stress inducers in L‐PDCKO male mice

**Figure 4:**
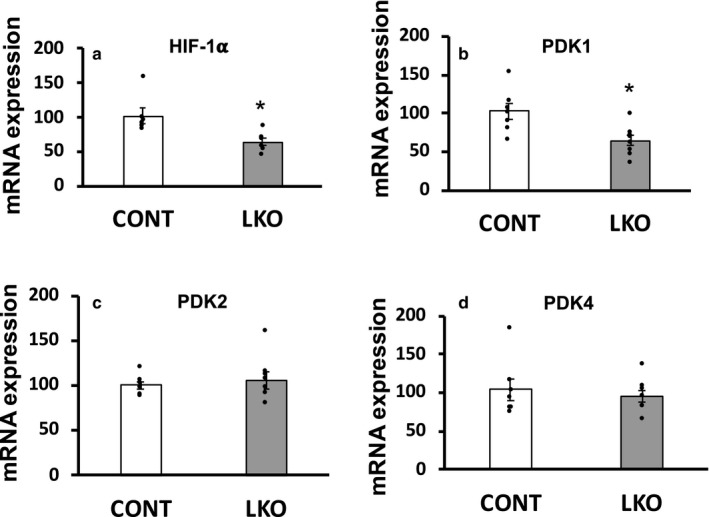
Quantitative real‐time PCR analysis of mRNA gene expression of (a) Hypoxia‐inducible factor‐1α (HIF‐1α; gene *Hif1α*), (b) Pyruvate dehydrogenase kinase, isoenzyme 1 (PDK1; *Pdk1*), (c) Pyruvate dehydrogenase kinase, isoenzyme 2 (PDK2; *Pdk2*), (d) Pyruvate dehydrogenase kinase, isoenzyme 4 (PDK4; *Pdk4*) in livers from control L‐PDCCT (CONT) and L‐PDCKO (LKO) mice. The results are means ±SE (*n* = 6–8). *p < 0.05

To evaluate if the observed “adaptations” in gene expression in the key enzymes in the glycolytic pathway were influenced by HIF‐1α and its downstream‐regulated *Pdk* genes, we analyzed expression of the *HIF*‐*1α* and *Pdk*1, 2 and 4 genes. Interestingly, expression of the *HIF*‐*1α* and *Pdk1* genes was significantly decreased whereas that of the *Pdk2* and *Pdk4* genes was not significantly altered in the liver of L‐PDCKO mice compared with L‐PDCCT mice (Figure 4a‐d).

**Figure 5:**
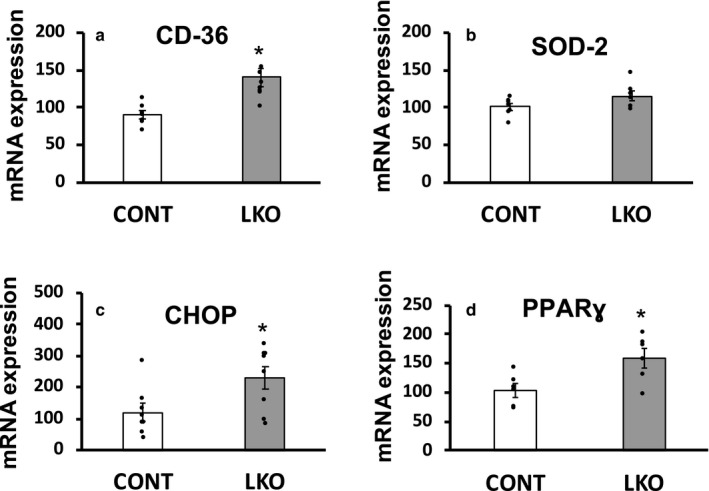
Quantitative real‐time PCR analysis of mRNA gene expression of (a) Cluster of Differentiation‐36 (CD36; gene *Cd36*), (b) Superoxide dismutase 2, mitochondrial (SOD2; *Sod2*), (c) CCAAT‐enhancer‐binding protein homologous protein (CHOP; *Chop*), and (d) Peroxisome proliferator‐activated receptor γ (PPARγ; *Pparg*) in livers from control L‐PDCCT (CONT) and L‐PDCKO (LKO) mice. The results are means ±SE (*n* = 6–8). *p < 0.05.

Because of a block in carbohydrate oxidative metabolism due to PDC deficiency, hepatocytes switch over to oxidation of alternate fuels and hence may experience metabolic and/or cellular stress. During the fasted state when glucose oxidation is inhibited due to PDC inactivation by its phosphorylation in the liver, long‐chain fatty acids are the preferred fuel for mitochondrial oxidative metabolism by hepatocytes. CD36 acts as a receptor for high affinity uptake of long‐chain fatty acids in tissues. Hence, we determined *Cd36* gene expression in the liver of L‐PDCKO mice. As seen in Figure 5a, *Cd36* gene expression was significantly up‐regulated in the liver of L‐PDCKO mice compared with L‐PDCCT mice.

PDC is localized in the mitochondrial matrix space, and hence its absence in hepatocytes may result in persistent oxidative stress. Since superoxide dismutase 2 (SOD2) is a key mitochondrial antioxidant, we evaluated its expression. Surprisingly, there was no significant alteration in *Sod2* gene expression in L‐PDCKO versus L‐PDCCT livers (Figure 5b) thereby suggesting that the former was not subject to excessive mitochondrial stress. In contrast, a significant increase in transcripts for C/EBP homologous protein (CHOP), an indicator of endoplasmic reticulum (ER) stress was observed in L‐PDCKO livers (Figure 5c).

Despite increased expression of several key glycolytic enzyme transcripts in the liver of L‐PDCKO mice, there is no evidence of increased proliferation and/or invasion (Jackson et al., 2017). Since peroxisome proliferator‐activated receptor γ (PPARγ) activation has been shown to inhibit the invasive and metastatic potential of hepatocellular carcinoma, we analyzed *Pparg* gene expression and found a significant increase in its expression in L‐PDCKO livers (Figure 5d).

**Figure 6:**
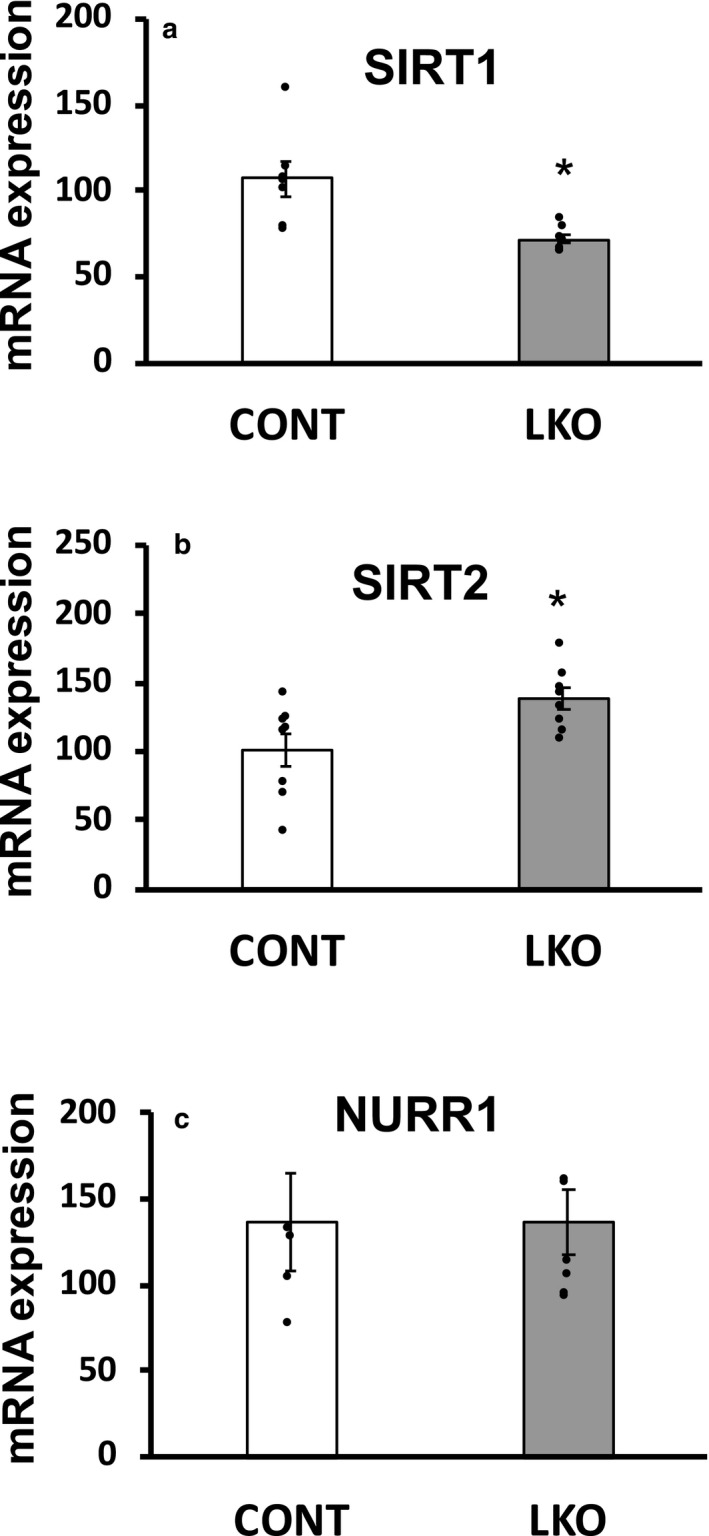
Quantitative real‐time PCR analysis of mRNA expression of (a) SIRT1 (gene *Sirt1*), (b) SIRT2 (*Sirt2*) and (c) NURR1(*Nurr1*) in livers from control L‐PDCCT (CONT) and L‐PDCKO (LKO) mice. The results are means ±SE (*n* = 5–8). * *p* < 0.05.

Sirtuin (SIRT)1 and 2 are histone/protein deacetylases and play important roles in cellular functions in both normal and cancer cells (Farcas et al., 2019). We found a significant reduction in *Sirt1* gene expression and an increase in hepatic *Sirt2* gene expression in L‐PDCKO mice compared with L‐PDCCT mice (Figure 6a & b). Increased expression of *Nurr1* is observed in some types of cancer. In this study, there was no significant change in *Nurr1* gene expression in the liver of L‐PDCKO mice compared with L‐PDCCT mice (Figure 6c).

## 4 DISCUSSION

**Figure 7:**
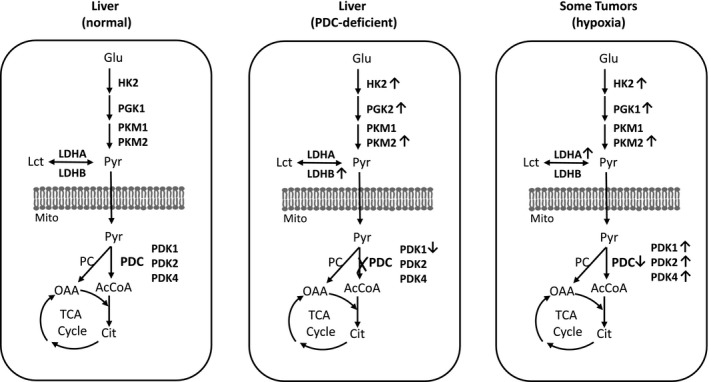
Schematic representation of the differences in several glycolytic gene expression in normal liver, liver deficient in PDC activity (due to *Pdha1* gene deletion), and some tumors in hypoxic conditions. Heavy X indicates genetic deletion of the *Pdha1* gene (and consequently absence of PDC functional activity) in hepatocytes. Direction of open arrows (up or down) next to enzymes indicates a significant change in gene expression in PDCKO liver and tumors in comparison to the respective genes in normal liver. Abbreviations: Mito, mitochondria; TCA Cycle, tricarboxylic acid cycle; Glu, glucose; Pyr, pyruvate; Lct, lactate; OAA, oxaloacetate; AcCoA, acetyl‐CoA; Cit, citrate; PC pyruvate carboxylase. Other enzyme/gene abbreviations as indicated in the text.

The functional utility afforded by “aerobic glycolysis” (aka Warburg effect) for some cancers is well‐documented (Goetzman & Prochownik, 2018; Vander Heiden et al., 2009). While inefficient in its energy generation process, it nevertheless provides cancer cells with increased supplies of intermediates needed for biosynthesis of macromolecules to support growth and proliferation. Aerobic glycolysis is enhanced by HIF‐1α, which up‐regulates the expression of many genes encoding glycolytic enzymes [such as HK2, phosphofructokinase 1 (PFK1), and phosphoglycerate kinase (PGK1) (Denko, 2008), GLUT1 and GLUT3 (Chen et al., 2001; Maxwell et al., 1997), PKM2 (Luo et al., 2011), LDHA (Keith et al., 2011), MCT4 (Keith et al., 2011; Ullah et al., 2006). HIF‐1α also decreases the transport of pyruvate (Eboli et al., 1977; Paradies et al., 1983; Schell et al., 2014) and its oxidation by PDC in the mitochondria, causing reduced oxidative respiration (Battello et al., 2016). In cancer cells and rapidly growing nontransformed cells with enhanced aerobic glycolysis, reduced pyruvate oxidation by PDC is achieved by at least five complimentary mechanisms: (a) posttranslational phosphorylation of specific α subunit tyrosine residues of PDH by oncogenic kinases [such as fibroblast growth factor receptor 1 (FGFR1)] causing inhibition of PDC activity (Fan et al., 2014; Hitosugi et al., 2011), (b) a novel posttranslational modification of pyruvate dehydrogenase phosphatase 1 (PDP1) by tyrosine phosphorylation by oncogene kinases to inhibit its activity (Shan et al., 2014), (c) increased transcription of *PDK1* to increase PDK1 activity to exert greater inhibition of PDH via serine phosphorylation of the α subunit, and (d) decreased levels of PDH and PDP2 (Jackson et al., 2017; Wang et al., 2019), and (e) the modulation of both PDK1 and PDP2 by small molecule products of oxidative phosphorylation such as ATP, NADH and acetyl‐CoA (Roche et al., 2001). The effects in (a) and (b) are exerted by oncogene kinases, and in (c) by HIF‐1α. These modulators, however, also regulate many other key genes or proteins responsible for increased aerobic glycolysis and oxidative phosphorylation (Wang et al., 2019). The findings presented here (Figure 7) show that the elimination of PDC activity in otherwise normal mouse liver by *Pdha1* gene deletion (without involving any cancer‐mediated modulators described above) is sufficient to induce Warburg‐type respiration as a result of PDC deficiency (Jackson et al., 2017; Reznik et al., 2017). In hypoxic cancer cells, increased expression of HIF‐1α induces gene expression of several key enzymes in the glycolytic pathway as well as it increases expression of pyruvate dehydrogenase kinase 1 (PDK1) which phosphorylates PDH and hence inhibits PDC activity (minimizing pyruvate oxidation to acetyl‐CoA and its further metabolism in the Krebs Cycle in the mitochondria). In the liver from normoxic L‐PDCKO mice, the expression of several genes (HKII, PKM2 and LDHB) is increased despite a decrease in the expression of HIF‐1α, suggesting that the mechanism responsible for induction of aerobic glycolysis between hypoxic tumors and livers from normoxic L‐PDCKO mice may differ to some extent. One limitation of this study is that it does not provide a direct support for increased aerobic glycolysis in PDCKO livers by increases in the carbon flux and/or in key enzyme activities in the glycolytic pathway.

We have previously observed no incorporation of [U‐^14^C]‐glucose‐derived^14^C‐carbon into fatty acids by liver slices from L‐PDCKO male mice (Choi et al., 2010). In a follow‐up study, down‐regulation of several key lipogenic genes, including (*Acc*, *G6pd2*, and *Hmgcr*) and their upstream regulators (SREB1c, SREBP2, ChREBP, LXR‐α, and *Pgc*‐*1α*) was observed in the livers from L‐PDCKO mice maintained in the fed state (Mahmood et al., 2016). This indicated that the bulk of *de novo* lipid synthesis requires pyruvate‐derived acetyl‐CoA and, in the absence of PDC, cannot be rescued by acetyl‐CoA derived from other sources. However, the mechanism(s) responsible for down‐regulation of these lipogenic genes remains obscure. The current findings also show that key glycolytic genes can be up‐regulated and drive Warburg‐type respiration in an otherwise normal tissue (mouse liver). It should be noted that within ~24 to 48 hr of fasting, hepatic PDC in normal rodents is nearly completely inactivated by serine phosphorylation with pyruvate being redirected into gluconeogenesis (Harris et al., 2002; Holness et al., 1987). This high degree of hepatic PDC inhibition during fasting, however, does not cause up‐regulation of any key glycolytic genes in liver from fasted mice. Contrarily, hepatic glycolysis is inhibited to support hepatic gluconeogenesis. It is possible that the time difference between the fasting period (24 to 48 hr only) and a prolonged adaptation in the livers of L‐PDCKO mice over several weeks may account, in part, for this phenomenon.

As described above, several distinct and nonmutually exclusive layers of transcriptional and/or posttranslational regulation permit enhanced activity of several key enzymes in the glycolytic pathway. In addition to PDH, these include HK2, PKM1 and 2, LDHA and B isozymes and PDKs (Prakasam et al., 2017; Xie & Simon, 2017). Expression of HK2 and PKM2 is low in normal adult mouse livers. Binding of PPARγ to the promoters of these two genes activates their transcription (Christofk et al., 2008; Kim et al., 2004; Panasyuk et al., 2012; Rempel et al., 1994). Expression of *Pparg* was increased in L‐PDCKO liver which may account for observed enhanced expression of HK2 and PKM2 in PDC‐deficient liver. Hong et al. (Hong et al., 2019) recently observed that transcriptional down‐regulation of LDHB (*Ldhb*) attenuated oxidative phosphorylation via lactate‐mediated modulation of the PDH/PDK axis in hepatoma cell lines. These investigators noted that a low LDHB/LDBA ratio was associated with enhanced glycolytic gene expression in cancer cells. Interestingly, expression of several glycolytic genes was increased in L‐PDCKO liver despite an increase in the LDHB/LDHA ratio, suggesting that this ratio may not be predictive of a change in glycolytic gene expression at least in noncancerous hepatocytes. It is noteworthy that livers from L‐PDCKO experienced a normoxic condition for availability of oxygen and hence it is expected that HIF‐1α expression will not be up‐regulated as it occurs in hypoxic tumor cells. We observed an increased expression of LDHB (and no change in LDHA expression) in L‐PDCKO livers unlike its down‐regulation of LDHB (and up‐regulation of LDHA) in hypoxic tumors. Low LDHB expression is associated with decreased oxidative phosphorylation activity via lactate‐mediated PDK‐PDH axis (Hong et al., 2019). In L‐PDCKO liver, increased expression of LDHB could support normal mitochondrial oxidative phosphorylation activity through fatty acid oxidation as indicated by increased expression of CD36 and PPARγ (Figure 5). Possible alterations in fatty acid oxidation and oxidative phosphorylation activity of mitochondria from L‐PDCKO mice remain to be investigated.

Mitochondrial PDC is regulated by PDKs (by phosphorylation and hence inactivation) and PDH phosphates (dephosphorylation and hence activation). Since the PDK/PDH axis is modified via increased expression of HIF‐1α in several tissue‐specific cancers, we quantified gene expression of three PDKs, and observed that the mRNA levels of HIF‐1a and PDK1 were significantly decreased whereas that of PDK2 and PDK4 remained unaltered in L‐PDCKO livers compared with L‐PDCCT livers (Figure 4). We did not measure expression of either mRNAs or enzymic activity of PDH phosphatases in livers of L‐PDCKO mice because any change in PDH phosphatases would not have any impact due to the absence of PDH protein.

Both observed increases and decreases in PDC activity in different cancers under hypoxic and normoxic conditions are consistent with the metabolic adaptations observed in these tumors (Chen et al., 2018; DeBerardinis et al., 2008; Denko, 2008; Dolezal et al., 2017; Ferriero et al., 2018). Cancer cells experiencing hypoxia‐induced PDC inhibition switch to aerobic glycolysis to enhance ATP generation with increased consumption of glucose with increased production of lactate (DeBerardinis et al., 2008; Goetzman & Prochownik, 2018; Vander Heiden et al., 2009).

In a recent study, compared to wild‐type rat fibroblasts, CRISPR/Cas9‐mediated *Pdha1*‐knockout rat fibroblasts showed a modest increase in glucose uptake with increased steady‐state levels of several glycolytic intermediates, notably pyruvate being very high without any significant change in lactate level under normal culture conditions (Wang et al., 2019). Interestingly, the levels of fructose and mannose‐6‐phosphate were also increased in PDH‐knockout rat fibroblasts, most likely derived from the selective glycolytic intermediates (Wang et al., 2019). PDH‐knockout rat fibroblasts showed lower oxygen consumption rates, indicating a greater dependence on glycolysis for ATP generation (Wang et al., 2019). Furthermore, there were significant changes in the steady‐state levels of the tricarboxylic acid cycle intermediates, a significant increase in oxaloacetate and reduction in the levels of the intermediates between α‐ketoglutarate to malate in PDH‐knockout rat fibroblasts compared with wild‐type fibroblasts (Wang et al., 2019). Unlike slowly proliferating liver cells in L‐PDCKO mice showing nearly 80% decrease in the level of acetyl‐CoA (Jackson et al., 2017), rapidly growing PDH‐knockout rat fibroblasts showed no significant change in acetyl‐CoA levels, indicating its origin from fatty acid oxidation and/or acetate (Wang et al., 2019). In this study we did not measure the concentrations of lactate and other glycolytic intermediates in support of aerobic glycolysis in L‐PDCKO livers.

We have previously reported that systemic null mutation as well as brain‐specific mutation in the *Pdha1* gene (located on chromosome X in mouse) was embryonic lethal for male embryos but allowed heterozygous female embryos to develop *in utero* and to develop/survive with about 50% reduction in PDC activity in all tissues (except the brain) (Johnson et al., 2001; Pliss et al., 2004, 2013). When tissue/cell‐specific *Pdha1* deletions were created [such as in pancreatic beta cells (Srinivasan et al., 2010), cardiomyocytes (Sidhu et al., 2008) and hepatocytes (Choi et al., 2010)], male embryos survived *in utero* and grew normally postnatally. As shown here, liver‐specific PDC deficiency is associated with the Warburg‐type respiration more characteristic of cancer cells (Figure 7). Whether this is a unique characteristic of liver metabolism or whether a similar aerobic metabolic switch develops in other tissue/cell‐specific PDCKO mouse models remains to be investigated. If it is unique to the liver, then it will be important to determine the specific determinant(s) that dictate the tissue specificity of this metabolic switch?

The mechanism(s) responsible for induction of aerobic glycolytic characteristic in noncancerous L‐PDCKO liver is not known at present. There are several inducers/modulators of the aerobic glycolysis switch in cancer cells, namely (a) HIF‐1α (Chen et al., 2001; Denko, 2008; Keith et al., 2011; Luo et al., 2011; Maxwell et al., 1997; Ullah et al., 2006), (b) specific oncogene kinases (Fan et al., 2014; Hitosugi et al., 2011), (c) tumor suppressors (Dang, 1999; Kamp et al., 2016), (d) the PI3 K/Akt/mTOR pathway (DeBerardinis et al., 2008; Elstrom et al., 2004; Fan et al., 2014; Hitosugi et al., 2011; Rathmell et al., 2003), (e) pH change due to lactate production (Hsu et al., 2016) and down‐regulation of mitochondrial oxidative phosphorylation via the PDK‐PDH axis (Hong et al., 2019), (vi) Sirtuins, and possibly others. As indicated earlier, the first three (a to c) inducers are not applicable to L‐PDCKO liver because of their noninvolvement in observed aerobic glycolysis in nontransformed L‐PDCKO liver. Increased Akt activity in the PI3 K/Akt/mTOR pathway (d) was found to be sufficient to induce the Warburg effect in both cancer as well as in nontransformed cells (Elstrom et al., 2004; Rathmell et al., 2003). A possible involvement of this signaling pathway in L‐PDCKO liver remains to be investigated. Similarly, increased lactate production (e) due to enhanced aerobic glycolysis would affect intracellular pH in L‐PDCKO hepatocytes. Cultured PDC‐deficient fibroblasts showed their cytoplasm to be more acidic and their mitochondrial matrix to be more alkaline (Wang et al., 2019). Whether a similar change in intracellular pH in L‐PDCKO liver is a cause or an effect is not known. The involvement of Sirtuins (f) is an attractive possibility for observed increased aerobic glycolysis in nontransformed PDC‐deficient hepatocytes. Wei et al. (2019) recently reported that SIRT1/MRPS5 axis is involved in metabolic reprogramming in liver cancer stem cells. The subcellular localization of mitochondrial ribosomal protein S5 (MRPS5) is determined by its acetylation status. Deacetylated MRPS5, resulting from increased SIRT1 expression, translocates to mitochondria to promote mitochondrial functions such as oxidative phosphorylation and generation of NAD^+^ (Wei et al., 2019). Low levels of SIRT1 maintain MRPS5 acetylation and permitting its nuclear translocation where it stimulates aerobic glycolysis (by yet noncharacterized mechanism) (Farcas et al., 2019; Wei et al., 2019). Interestingly, in this study SIRT1 expression was decreased in L‐PDCKO hepatocytes, keeping more of MRPS5 in acetylated form and promoting its translocation to the nucleus to enhance the Warburg effect. SIRT2 up‐regulation has been reported in primary hepatocellular carcinoma tumors and correlated with shorter patient survival (Huang et al., 2017). SIRT2 expression was also increased in L‐PDCKO liver. Unlike SIRT1, SIRT2 did not deacetylate MRPS5 (Wei et al., 2019).

Finally, in some cancer cells in which PDHα protein level and PDC activity are increased (Chen et al., 2018; Dolezal et al., 2017; Marin‐Valencia et al., 2012) the supply of mitochondrial acetyl‐CoA for increased *de novo* biosynthesis of lipids as well as protein acetylation in the cytoplasmic/nuclear compartment is supported for rapid growth (Dolezal et al., 2017; Marin‐Valencia et al., 2012). In contrast, when PDC activity is inhibited due to the increased PDK activity in some cancer cells, the supply of pyruvate‐derived acetyl‐CoA in the form of citrate from mitochondria to the cytosolic/nuclear compartment is inhibited. In such cases, mitochondrial PDC may translocate to the nucleus (Sutendra et al., 2014), allowing a more efficient and/or directed generation of acetyl‐CoA that is dedicated solely to histone acetylation and gene regulation in the nucleus (Boukouris et al., 2016; Chen et al., 2018; Ferriero et al., 2018). The deletion of the *Pdha1* gene resulted in a marked reduction in the acetyl‐CoA level in L‐PDCKO liver (Jackson et al., 2017). Hence, its impact on protein acetylation in the nucleus and its effects on gene transcription of key enzymes in the glycolytic pathway remains to be investigated.

## CONFLICT OF INTEREST

The authors declare that there are no conflicts of interest, financial or otherwise, for this study.

## AUTHORS’ CONTRIBUTIONS

M.S.P.: conception and financial support; M. S. P., S. M., and T.C.R.: design of research and interpretation of results; S.M. and J. J. performed the experiments and prepared the figure; M.S. P. drafted the manuscript; All contributed to revision of the manuscript and approved the final version of manuscript.

## Supporting information



Supplementary Material
