# Corrigendum: Reprogramming of aerobic glycolysis in non‐transformed mouse liver with pyruvate dehydrogenase complex deficiency

**DOI:** 10.14814/phy2.14793

**Published:** 2021-03-05

The authors of the article by Patel et al. (2021) noticed an error in the funding information. The presently listed information in the paper is: Funding information: This work was supported in part by US Public Health Service Grant NS093264 (MSP).

However, the correct information should read as:

Funding information: This work was supported in part by US Public Health Service Grant DK‐20478 (MSP).
