# Supplementary material for: Reprogramming of aerobic glycolysis in non‐transformed mouse liver with pyruvate dehydrogenase complex deficiency
Source: Physiol Rep. 2021 Jan 5;9(1):e14684. doi: 10.14814/phy2.14684 (PMC7785054; doi:10.14814/phy2.14684)
Supplement: Supplementary file 1 — Supplementary Material [file PHY2-9-e14684-s001.docx]

| Gene name | Gene symbol | Direction | Primer sequence | Product size (bp) |
| --- | --- | --- | --- | --- |
| Hexokinase 2 | *Hk2* | Forward | 5’-TGATCGCCTGCTTATTCACGG-3’ | 112 |
|  |  | Reverse | 5’-AACCGCCTAGAAATCTCCAGA-3’ |  |
| Phosphoglycerate kinase 1 | *Pgk1* | Forward | 5’-AACCTCCGCTTTCATGTAGAG-3’ | 120 |
|  |  | Reverse | 5’-GACATCTCCTAGTTTGGACAGTG-3’ |  |
| Enolase 1 | *Eno1* | Forward | 5’-TGCGTCCACTGGCATCTAC-3’ | 118 |
|  |  | Reverse | 5’-CAGAGCAGGCGCAATAGTTTTA-3’ |  |
| Pyruvate kinase 1 | *Pkm1* | Forward | 5’-GCTGTTTGAAGAGCTTGTGC-3’ | 87 |
|  |  | Reverse | 5’-TTATAAGAGGCCTCCACGCT-3’ |  |
| Pyruvate kinase 2 | *Pkm2* | Forward | 5’-TCGCATGCAGCACCTGATT-3’ | 65 |
|  |  | Reverse | 5’-CCTCGAATAGCTGCAAGTGGTA-3’ |  |
| Lactate dehydrogenase A | *Ldha* | Forward | 5’-GCTCCCCAGAACAAGATTACAG-3’ | 131 |
|  |  | Reverse | 5’-TCGCCCTTGAGTTTGTCTTC-3’ |  |
| Lactate dehydrogenase B | *Ldhb* | Forward | 5’-AGTCTCCCGTGCATCCTCAA-3’ | 101 |
|  |  | Reverse | 5’-AGGGTGTCCGCACTCTTCCT-3’ |  |
| Pyruvate dehydrogenase kinase, isoenzyme 1 | *Pdk1* | Forward | 5’-TCCCCCGATTCAGGTTCAC-3’ | 65 |
|  |  | Reverse | 5’-CCCGGTCACTCATCTTCACA-3’ |  |
| Pyruvate dehydrogenase kinase, isoenzyme 2 | *Pdk2* | Forward | 5’-CACCGGACTCTAAGCCAGTT-3’ | 122 |
|  |  | Reverse | 5’-ACGGGGTCATCTCCATAGGT-3’ |  |
| Pyruvate dehydrogenase kinase, isoenzyme 4 | *Pdk4* | Forward | 5’-GATTGACATCCTGCCTGACC-3’ | 98 |
|  |  | Reverse | 5’-CATGGAACTCCACCAAATCC-3’ |  |
| Hypoxia inducible factor 1, alpha subunit | *Hif1a* | Forward | 5’-ACCTTCATCGGAAACTCCAAAG-3’ | 228 |
|  |  | Reverse | 5’-CTGTTAGGCTGGGAAAAGTTAGG-3’ |  |
| Cluster of differentiation 36 | *Cd36* | Forward | 5’-TGGCTAAATGAGACTGGGACC-3’ | 124 |
|  |  | Reverse | 5’-ACATCACCACTCCAATCCCAAG-3’ |  |
| Superoxide dismutase 2, mitochondrial | *Sod2* | Forward | 5’-ATCTGTAAGCGACCTTGCTC-3’ | 228 |
|  |  | Reverse | 5’-GCCTGCACTGAAGTTCAATG-3’ |  |
| DNA damage-inducible transcript 3 | *Chop* | Forward | 5’-CTGGAAGCCTGGTATGAGGAT-3’ | 121 |
|  |  | Reverse | 5’-CAGGGTCAAGAGTAGTGAAGGT-3’ |  |
| Peroxisome proliferator activated receptor gamma | *Pparg* | Forward | 5’-TGTGGGGATAAAGCATCAGGC-3’ | 120 |
|  |  | Reverse | 5’-CCGGCAGTTAAGATCACACCTAT-3’ |  |
| Silent information regulator 1 | *Sirt1* | Forward | 5’-AGAACCACCAAAGCGGAAA-3’ | 122 |
|  |  | Reverse | 5’-TCCCACAGGAGACAGAAACC-3’ |  |
| Silent information regulator 2 | *Sirt2* | Forward | 5’-AGCCAACCATCTGCCACTAC-3’ | 194 |
|  |  | Reverse | 5’-CCAGCCCATCGTGTATTCTT-3’ |  |
| Nuclear receptor related 1 protein | *Nurr1* | Forward | 5’-CAACTACAGCACAGGCTACGA-3’ | 98 |
|  |  | Reverse | 5’-GCATCTGAATGTCTTCTACCTTAATG-3’ |  |

Supplemental Table 1: Primer sequences used for quantitative real-time PCR analysis
